# Diabetes Self-Management in the Age of Social Media: Large-Scale Analysis of Peer Interactions Using Semiautomated Methods

**DOI:** 10.2196/18441

**Published:** 2020-06-30

**Authors:** Sahiti Myneni, Brittney Lewis, Tavleen Singh, Kristi Paiva, Seon Min Kim, Adrian V Cebula, Gloria Villanueva, Jing Wang

**Affiliations:** 1 University of Texas School of Biomedical Informatics at Houston Houston, TX United States; 2 Center on Smart and Connected Health Technologies School of Nursing The University of Texas Health Science Center at San Antonio San Antonio, TX United States

**Keywords:** diabetes, self-management, social media, digital health

## Abstract

**Background:**

Online communities have been gaining popularity as support venues for chronic disease management. User engagement, information exposure, and social influence mechanisms can play a significant role in the utility of these platforms.

**Objective:**

In this paper, we characterize peer interactions in an online community for chronic disease management. Our objective is to identify key communications and study their prevalence in online social interactions.

**Methods:**

The American Diabetes Association Online community is an online social network for diabetes self-management. We analyzed 80,481 randomly selected deidentified peer-to-peer messages from 1212 members, posted between June 1, 2012, and May 30, 2019. Our mixed methods approach comprised qualitative coding and automated text analysis to identify, visualize, and analyze content-specific communication patterns underlying diabetes self-management.

**Results:**

Qualitative analysis revealed that “social support” was the most prevalent theme (84.9%), followed by “readiness to change” (18.8%), “teachable moments” (14.7%), “pharmacotherapy” (13.7%), and “progress” (13.3%). The support vector machine classifier resulted in reasonable accuracy with a recall of 0.76 and precision 0.78 and allowed us to extend our thematic codes to the entire data set.

**Conclusions:**

Modeling health-related communication through high throughput methods can enable the identification of specific content related to sustainable chronic disease management, which facilitates targeted health promotion.

## Introduction

### Background

Diabetes (specifically type 2 diabetes and prediabetes) is a leading public health burden and global health issue. As of 2019, more than 100 million US adults are now living with diabetes or prediabetes [1]. The total estimated cost of diagnosed diabetes in 2020 is $327 billion, including $237 billion in direct medical costs and $90 billion in reduced productivity [1]. Individuals with diagnosed diabetes have annual medical expenditures that are $7900 or approximately 2.3 times higher than they would be in the absence of diabetes ($13,700 vs $5800) [2]. Diabetes can also lead to renal and cardiovascular complications [1]. Addressing lifestyle risk factors, such as poor diet and physical activity, is vital to diabetes prevention and management. Numerous interventions and public health campaigns have been developed to help patients incorporate new behaviors (eg, medication regimen) and modify existing risky behaviors (eg, poor diet) to prevent and manage diabetes (for reviews, see [3-7]). However, the growth rate of diabetes is steady, adding to the health care burden. Adherence to healthy behaviors (eg, proper nutrition) and management of prevailing health conditions (eg, medication adherence) requires a significant support infrastructure that targets individualistic factors and environmental influences for long time intervals [8,9].

### Social Relationships and Health Management

Recent research suggests that social relationships play an essential role in an individual’s engagement in health issues [10-12]. For example, Christakis and Fowler’s analysis of the Framingham data set shows an association between the behavior of members of an individual’s social network and the likelihood of smoking cessation [13]. Positive effects of social relationships have been associated with chronic illness self-management [14-17]. Increased levels of social integration are also found to improve the overall wellbeing of individuals [18]. On the other hand, some studies indicate the negative influence of social relationships [19,20]. While community-based social interventions harnessing the positive effects of social contacts exist [21-24], the mechanisms underlying the impact of social relationships on multiple behavioral domains of Diabetes Self-Management (DSM) are not fully understood. Consequently, an understanding of the mechanisms in play for numerous behavioral domains within diabetes management is crucial to promote wellness regimens that can result in sustained adoption.

### Online Communities as Secondary Data Sources

The ubiquity of online communities presents us with invaluable data sets in the form of electronic traces of peer interactions [25], which may help to understand social influence in diabetes management. Thanks to the ready availability and accessibility of the internet via mobile phones, peer interactions in online communities often occur in real time. They can provide rich documentation of certain crucial moments in everyday life that influence diabetes prevention and management [26]. Further, it is common for an individual to seek a related online community (eg, newly diagnosed with type 2 diabetes) and navigate the records of peers who have shared their experiences. With the support of online communities and an associated bank of collective knowledge, the individual reflects on the problem, explores available information, and feels able to act, thus eliciting multiple theoretical constructs described in existing models of behavior change ([27-31]. Emerging research shows the complex relationships between online social ties and individuals’ self-management of health conditions, thus highlighting the utility of online peer interactions as secondary data sources [17,29]. While we must be cognizant of inferential generalizability [30], these platforms have a tremendous capacity to inform clinicians, behavioral scientists, and technology developers about human health behaviors and ways to harness knowledge from online social media to inform intervention design, content curation, and information dissemination [31-34]. A more in-depth analysis of such interactions provides us with a new lens to inform, enhance, and strengthen existing frameworks of diabetes care delivery, prevention, and management [29,31]. Previous studies on diabetes-related social media interactions have focused on general-purpose platforms such as Twitter and Facebook interventions, where data volume has ranged in the order of hundreds to billions [35-39]. A majority of these studies have attempted to understand the types of diabetes information disseminated, the levels of information spread, and user engagement facilitated by these platforms. However, our understanding of digital environments solely dedicated to diabetes prevention and self-management are quite limited. As such, the semantic context underlying general-purpose and health-specific platforms can vary greatly, consequently affecting the methodological underpinnings of large-scale studies for unpacking the DSM domain in social media.

In this paper, we describe our findings of large-scale analysis of peer interactions in the health-related online community focusing on diabetes management. In addition to abstracting thematic strands underlying peer interactions, we provide a more in-depth analysis of behavior change techniques that manifest in these online discussions using manual coding methods. Further, we extend the reach of qualitative analysis using high throughput computational methods to understand the thematic distribution of peer communication in a diabetes-specific online community. The insights gained from these investigations will enable us to gain a deeper understanding of the digital environment and the nature of the peer interactions they facilitate, inclusive of and beyond social support. Our findings will help us design an enhanced support infrastructure through the development of tailored education interventions and digital solutions that harness social support and influence to promote positive health changes. Such “healthier life” technologies offer considerable advantages over traditional approaches in affordability, scalability, user engagement, and personalization.

## Methods

### Materials

For this study, we focus on user interactions within the American Diabetes Association (ADA) online community, one of the largest online communities focusing on engaging patients with diabetes and their caregivers in optimizing self-health management [40]. Members are required to have a registered account with the ADA to share content and exchange messages within the online community. The data set spans eight years (2012-2019) and includes publicly available interactions. Behavior before and after diagnosis, treatment effectiveness, healthy behaviors (low carb diet, physical activity), medication adherence, blood glucose self-monitoring, and other topics are discretely captured in this data. For this project, we focused our analysis on type 2–related entries. A total of 80,481 randomly selected de-identified messages exchanged by 1212 members were included in this analysis. We chose type 2 diabetes as the focus of this study because health outcomes and disease management among these patients are impacted by their lifestyle behaviors (diet and physical activity), medication use, and self-monitoring of blood glucose. The research has been reviewed and exempted by the Institutional Review Board at the University of Texas Health Science Center at Houston.

### Theme Abstraction

We adopted Directed Content Analysis [41] to identify the core concepts and unifying themes that relate to diabetes prevention and management. First, four independent coders characterized the communication between members of each community, assigning communication themes (inductively derived using grounded theory techniques [42] in our prior work [43]) to randomly selected messages that relate to diabetes prevention and management. Table 1 provides an overview of the qualitative analysis and coding categories. We coded 517 messages to assign thematic labels (shown in Table 1). Each message could have multiple codes applied dependent on the content of the message, and codes were individually and independently assigned by four coders. Each message will have a minimum of two independent coders applying codes. Coders then met and reconciled codes into a master coded document via weekly meeting discussion following iterative comparison and consensus building to ensure objectivity in the coding process. The qualitative analysis allowed us to explain how online platforms are utilized by individual users to mend the gaps in their social and information needs. Also, we conducted a more in-depth analysis of the messages to understand types of social support [44] and the taxonomy of behavior change techniques [45] observed in peer interactions.

**Table 1:** Sample messages from the American Diabetes Association (ADA) mapped to the communication themes.

Theme	Definition	Sample message snippets from ADA
Social support	Messages where the content reflects the elements of praise, advice, empathy, and guidance	Congratulations on a job well done – and Welcome to the 5% club. Your hard work and persistence paid off. Keep it up. :)
Traditions	Messages that focus on community-specific rituals such as pledges or any engagement practices conducted by moderators or users	How did you do this morning? How’ve you been doing over time? Nobody knew back then that there would be 28,196 replies to … question. Nobody expected that twice that topic would grow so large that we would have to start over again in a brand new topic to accommodate all those posts.
Teachable moments	Messages that describe incentives to make positive health changes	Stress can have a huge impact on your numbers. Even a single day can raise my numbers significantly and I have had longer periods of stress that I know upped my A1C. So when you are dealing with a stressful time you want to increase your exercise and decrease your carbs.
Obstacles	Messages focusing on hurdles to planned health practices	I did add 3 days of swimming that lasted for 3 months until my swim buddies got on different schedules. I do miss the sun and water so I’m on a search for other swimming holes and buddies. Transportation can be a hurdle, too.
Pharmacotherapy	Messages with explicit discussions on various pharmacotherapy options	Metformin may have a small effect reducing insulin resistance, but its main effect is to keep the liver from sending out too much insulin and over-compensating when blood glucose is a little low, like when it helps to prevent the dawn effect.
Relapse	Messages with descriptions of relapse reasons or confessions	On the issue of my numbers being too high in general… that’s a separate issue. I have gotten lax with exercise and eating too many carbs.
Readiness to change	Messages that inspire to initiate positive health changes	I discovered that I had to change “Can’t” to “Don’t” in my thinking. I “can’t” eat that cookie… means “Poor me, someone… is not allowing me to eat that cookie”… I “don’t” eat cookies… means that I have a choice it’s not something that’s part of my life. I am in control.
Cravings	Messages that capture real-time expressions of the urges to deviate from planned health behaviors	Do I miss stuffing my face with pizza or other carbilicious meals? I suppose so, but it’s not much of a loss… I miss sugary snacks, I guess that the biggest change.
Alternative medicine	Messages that describe therapies that are not regarded as orthodox by the medical profession	The article has a story of one woman who was getting ready to have a foot/leg amputated (after living with “a terrible wound for 5 years”), but she tried ‘the sugar treatment’ (my term) and … She ended up not having an amputation.
Progress	Messages in which members communicate their progress based on objective health measures	This summer will mark 8 years since I have been diagnosed with Type 2 diabetes. So far low carb eating, exercise and metformin are keeping me at my target blood glucose numbers.
Patient-reported Outcomes	A message that focuses on subjective progress (positive or negative)	Do I sometimes want to go back? Yes and no. I feel much better now and I know I’m healthier now, so no, I don’t want to go back.
Conflict	A message which is argumentative or clarifying a point/topic (not necessarily supportive)	Again I did not say it causes diabetes I said it can cause diabetes – which was the original question. I did not say that there is a direct link between alcoholism and diabetes – but the actions of an alcoholic can contribute to developing diabetes.
Miscellaneous	A message which contains questions or information not about an individual’s health status or diabetes management	I’m almost done with my First semester of college. Can you believe that? I did lot hard work.

### Automated Methods

Vector representations of all 80,481 messages were generated using distributional semantics methods [46]. The entire data set was then annotated by using the generated vectors as input to a machine learning classifier trained on the manually annotated messages. We exploited recent developments in automated text analysis to measure the extent to which key concepts of interest were expressed within messages between ADA community users, regardless of the specific terms used to express these concepts at the surface level. We applied latent semantic analysis [47], a method of distributional semantics in conjunction with a machine-learning classifier to derive a measure of relatedness between a given message and the previously identified communication themes to estimate the distribution of different types of content across the ADA online community. Ten-fold cross-validation was applied to determine the best performing binary classifier for automating the classification of the entire set of messages. We have used Weka [48] and Semantic Vectors package [49] to build the pipeline for automated classification of ADA peer interactions.

## Results

### Qualitative Analysis

#### Theme Abstraction

Based on manual coding of 517 messages, “Social support” was the most common comment theme (n=439, 84.9% of comments), followed by “readiness” (n=97, 18.8%), “teachable moments” (n=76, 14.7%), “pharmacotherapy” (n=71, 13.7%), and “progress” (n=69, 13.3%), “obstacles” (n=48, 9.2% of comments). Additional codes included, “miscellaneous” (n=33, 6.3%), “patient-reported outcomes” (n=29, 5.6%), “traditions” (n=25, 4.8%), “conflict” (n=24, 4.6%), “alternative medicine” (n=7, 1.3%), “relapse” (n=5, 0.97%), and “cravings” (n=1, 0.19%). Given the very nature of the social forum, the majority of the messages exchanged in the ADA community were fostering empathy, affection, and reinforcement that are essential to the sustenance of healthy lifestyle changes. Medication use, motivators for change, and sharing progress also seem to play an important role in diabetes interactions in this community.

#### Social Support—Anatomical Analysis

A more in-depth analysis of messages specific to social support theme using House taxonomy [39] revealed that the most common form of social support provided was “informational” (n=361, 82.2%), followed by “emotional” (n=155, 35.3%), and “appraisal” (n=9, 0.02%). “Instrumental” support did not apply to our data set, given the lack of manifestation of tangible support (Table 2).

Further analysis revealed the specific behavior change techniques employed by ADA community users. “Social Support,” “Shaping knowledge,” “Feedback and Monitoring,” and “Goals and Planning” were the most utilized behavior change techniques embedded within the messages related to social support theme.

**Table 2:** Social support analysis.

Types of social support	Definition	Example
Informational	Providing advice, suggestions, and information	“I wait for about 6-7 days of bg readings to call a trend for myself when the differences are small, but it is possible over a course of days to note a slight uptick or downtick in bg.”
Emotional	Expressions of empathy, love, trust, and caring	“Way to go …! Congratulations on changing your way of eating and adding in all that exercise.”
Appraisal	Information that is useful for self-evaluation	“Did you ever have diabetes education classes, or consult with a diabetes educator? Do you know how to count carbs? Read here and learn how to make your efforts achieve the best possible outcomes.”
Instrumental	Providing tangible aid and service	N/A^a^

^a^N/A: not applicable.

#### Beyond Social Support—Anatomical Analysis

**Figure 1:**
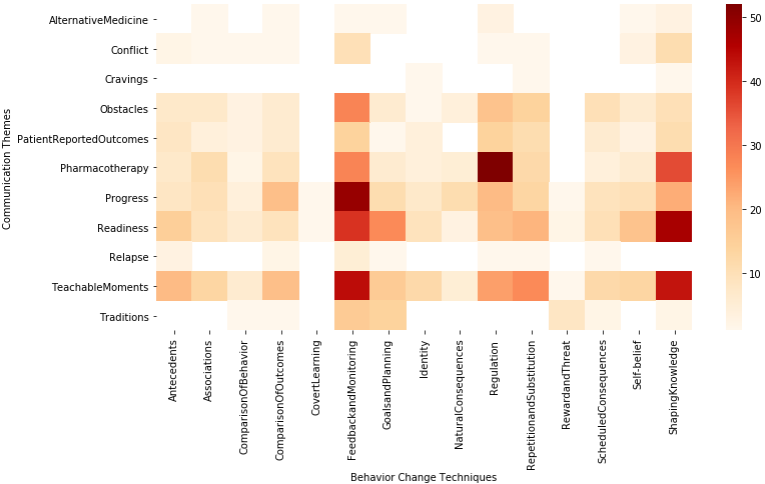
Mapping of communication themes and behavior change techniques.

Figure 1 shows the thematic dispersion (excluding “Social Support”) across various behavior change techniques, where the color scale represents the number of messages in which a given technique has been observed. “Feedback and monitoring” was the most diversely used technique, followed by “Shaping knowledge,” “Goals and Planning,” and “Repetition and substitution,” and “Regulation.” The least used behavior change techniques include “covert learning,” “rewards and threat,” and “natural consequences.”

### Automated Classification

The precision, recall, and f-measure for the cross-validation of the machine learning technique using the SVM classifier were 0.76, 0.78, and 0.77, respectively. Table 3 provides a summary of the performance for the most commonly used classifiers.

Due to insufficient training examples in the training set, we disregarded 5 of the 13 themes for final classification. Due to a lack of semantic context, we have not included “miscellaneous” in our automated classification system. With the application of our automated classification to the rest of the ADA data set (n=80,481 posts), thematic coverage is as follows: social support (74.2%), readiness to change (12.6%), progress (18.8%), obstacles (10.2%), teachable moments (16.4%), Pharmacotherapy (21.4%), and Patient-reported outcomes (7.1%).

Given the use of high throughput analytical methods to extend manual coding to the rest of the ADA data set, we were able to gain an understanding of the prevalence of DSM-related communication themes in this online community. Understanding thematic prevalence at large scale will now help us with the development of automated support systems using virtual coaching and chatbots for seamless and sustained user experience in online communities such as ADA.

**Table 3:** Machine learning classifiers applied to peer interactions.

Naïve	Recall	Precision	F-measure
LibLinear	0.64	0.66	0.65
SVM^a^	0.76	0.78	0.77
KNN^b^	0.68	0.65	0.66
J48	0.72	0.66	0.69
Naïve Bayes	0.78	0.54	0.64

^a^SVM: support vector machine.

^b^KNN: k-nearest neighbors.

## Discussion

### Principal Findings

In this digital era of connected health consumers, the interplay between theory-driven models of diabetes management and observed communication in social media is currently poorly understood [50]. Previous studies have shown that those with DSM who participate in social media forums or platforms saw a decrease in their HbA_1c_ (glycated hemoglobin) [51]. In the future, physicians may “prescribe” a form of social media or platform to reinforce healthy lifestyle choices outside of the clinic.

The results of this study facilitate the ecological analysis of DSM as embedded in peer interactions. This analysis may warrant refining existing models of DSM in the context of face-to-face (rather than online) communication. By using automated social media analysis methods, we will be able to scale up the qualitative analysis to extract relevant communication from large online social media data sets. Though analysis of diabetes management in online health communities is not without precedent [52], prior research does not address methodological scalability and shortcomings to model variances in multiple behaviors and underlying communication attributes in social settings. In this research, we conducted an inductive analysis of DSM strategies, without reliance on a single behavior change theory, as embedded in communication exchanges among members of a health-related online community. This effort enables the extraction of information context significant to behavior change events and social engagement levels in self-management of health-related activities.

Frequent use of online networks for social support, mainly informational, indicates a possible need for individualized diabetes support personnel outside of physician offices. It was noted that users would turn to the online forum to develop a consensus regarding the effectiveness of their medication regimen, exercise routines, and nutritional needs of people with diabetes. A minority of the comments provided solely emotional social support and many comments offered anecdotes to provide context for their diabetes journey. The online forum is a potential method of distributing information regarding their specific illness and sharing new recommendations, as users often share articles and studies they see as relevant or personal experience that helped them better manage their diabetes.

Current research on diabetes prevention and self-management has not addressed the effects of information and social environment. Prior work on content-inclusive network analysis [53-55] provided new methods for modeling network diffusion of communication attributes in online health communities, thus enabling us to disentangle the effects of the theoretical properties of exchanged health information and social structure on health outcomes. With the onset of mobile connectivity in the communication sector, messages exchanged in health-related online communities reflect the intricacies of human health as experienced in real time at the individual, community, and societal levels [33]. The majority of research studies on online health communities focusing on diabetes have analyzed peer-to-peer interactions based on social support categories facilitated by the platforms (eg, informational support, emotional support) [56-58]. However, social support is but one of the numerous interpersonal mechanisms facilitated by the social ties established in online communities. Existing theories of behavior change suggest a myriad of content-driven strategies to elicit specific socio-behavioral mechanisms beyond social support (eg, stimulus control, observational learning) to help individuals change their behavior and self-manage an illness [43,59,60]. Our qualitative analysis of underlying behavior change techniques in peer interactions has highlighted “feedback and monitoring” to be the most used technique, which emphasizes the complex functions of social relationships, which goes beyond the provision of social support. ADA-like platforms can help provide better self-health awareness for individuals through monitoring and knowledge acquisition.

### Limitations and Future Work

Our qualitative coding has been limited to inductive analysis and mapping of behavior change techniques in a single online community. Future research should focus on mapping of these inductively derived themes to expansive theory-driven taxonomy such as the Behavior Change Taxonomy [45,60] using computational models for large-scale pattern recognition and identification of independent behavior strands within the DSM in online settings. Further, there may be differences in what is gained from using social media platforms like ADA based on user demographics. Future studies should consider age-specific barriers to information consumption and comprehension in social media platforms. Although we used multiple computational models to perform a large-scale analysis of ADA user interactions, the use of advanced deep learning methods from artificial intelligence research, such as Convolutional Neural Networks [61] and Bidirectional Encoder Representations from Transformers [62], may improve the training of the automated classification system.

Further analysis of peer communication can be deepened through sentiment analysis to find specific emotions in communication, such as anger, happiness, and others. Quantifying sentiments [63] can also help in differentiating their sentiments towards the interventions or other aspects of the behavior change process and regimen. This effort will, in turn, help interventionists identify attitudes and further motivation for user engagement that can arise from satisfaction/dissatisfaction with the intervention.

### Conclusions

Behavior modification, such as balanced nutrition, an increase in physical activity, and medication adherence, is a critical component of DSM. Patient engagement in DSM consists of the adoption of healthy behaviors and abstinence from risky behaviors. However, the modification of such behaviors is challenging. Numerous public health efforts have been made to promote healthy behaviors over the years, but their utility and efficacy have been suboptimal. The utility of online social media to foster behavior change has been recognized as one sustainable solution. However, little is known about how we can harness social platforms to facilitate positive changes and promote DSM. Health-related online communities present a unique opportunity to improve our understanding of such socio-behavioral mechanisms, as communication in this context is digitally archived, permitting analysis of the dynamics of social influence as they manifest in peer interactions. Our methods have allowed us to abstract the essence of peer-to-peer communication in online communities at scale and to elucidate ways in which observable digital interactions relate to behavior modification endeavors as related to diabetes prevention and management. Our findings will provide the basis for an integrated approach to the problem of chronic disease management and underlying subtasks of behavior change. Such work will have implications for the design of behavior support technologies that offer automated personalization to improve self-management behaviors at the individual and population level.

## Abbreviations

ADA: American Diabetes Association
DSM: diabetes self-management
HbA_1c_: glycated hemoglobin
SVM: support vector machine
KNN: k-nearest neighbors
